# Beyond KCH selection and options in acute liver failure

**DOI:** 10.1007/s12072-018-9869-7

**Published:** 2018-06-01

**Authors:** William Bernal, Roger Williams

**Affiliations:** 1 The Institute of Liver Studies, Cheyne Wing, King’s College Hospital, Denmark Hill, London, SE5 9RS UK; 2 The Institute of Hepatology London and Foundation for Liver Research, 111 Coldharbour Lane, London, SE5 9NT UK; 3 Faculty of Life Sciences & Medicine, King’s College, London, UK

**Keywords:** Acute liver failure, King’s criteria, Dynamic model, ALFSG, Plasma exchange, Liver transplant

## Abstract

The King’s prognostic criteria for patients with acute liver failure (ALF) introduced in 1989 have been used worldwide. This distinguished for the first time cases with ‘hyper-acute’ course (characteristically paracetamol overdose) where there is a better chance of recovery with medical supportive care alone from those etiologies with a less acute course and paradoxically lower chances of ‘spontaneous’ recovery. Ongoing use showed the limited sensitivity of the criteria to constitute a significant practical limitation. Subsequent models including the MELD score and composite ones with markers of necrosis, an apoptotic liver cell death, proposed to improve sensitivity did not have the required high specificity. Two recent models utilizing new availability of web- and app-based computing delivering outcome predication through sophisticated algorithms are described. The first is a dynamic model described for paracetamol-induced ALF based upon admission findings and sequential variables over the first 2 days. The new model of the US Acute Liver Failure group was devised to cover all etiologies of ALF for predicting ‘transplant-free’ survival and accurately predicated spontaneous survival in two-thirds of cases. Improved survival results with medical management, particularly in hyper-acute cases, now approach those obtained with successful liver transplant and have raised the question of transplant benefit. Also considered in the review are new non-transplant approaches to treatment including the use of plasma exchange and based on successful results in acute-on-chronic liver failure, agents to modulate and improve hepatic regeneration.

## Introduction and historical context

Difficulties in determining prognosis in patients with acute liver failure (ALF) from paracetamol hepatotoxicity have been apparent for many years. Initial issues related to the difficulties in identifying those patients with paracetamol intoxication who would benefit from antidotal therapy [1]. The need for accurate predictive tests of patient outcome became even more apparent when the life-saving procedure of liver transplant for patients with ALF was introduced in the early 1980. Concerns were raised that some patients would be transplanted who would otherwise have recovered with medical management alone; variously termed ‘transplant-free’ or ‘spontaneous’ survival.

The description of the King’s College Criteria (KCC) in 1989 represented a major step forward in identifying candidates for liver transplantation (Text Box 1) [2]. It stressed the importance of etiology and mode of presentation in the outcome of ALF, recognizing the chances of recovery with medical supportive care alone with rapidly evolving paracetamol hepatotoxicity as being much higher than with other etiologies of ALF, particularly those with illness of more gradual onset. Derived and validated in a cohort of 763 patients managed before the introduction of transplantation for ALF, the high specificity of the KCC meant that relatively few cases fulfilling criteria would be unnecessarily transplanted. However, their sensitivity was lower, indicating that a significant number of cases not fulfilling criteria would progress and die without earlier identification and consideration for possible liver transplantation. For the non-paracetamol cases, both sensitivity and specificity were high but within this overall group, certain etiologies with rapid illness onset—specifically ischemic hepatitis, acute fatty liver of pregnancy and hepatitis A—had similar survival rates with medical management alone to that of paracetamol-induced cases. It was in those cases with an indolent presentation and ‘sub-acute’ phenotype and often ‘indeterminate’ etiology that had the worst outcome without transplantation [3]. 
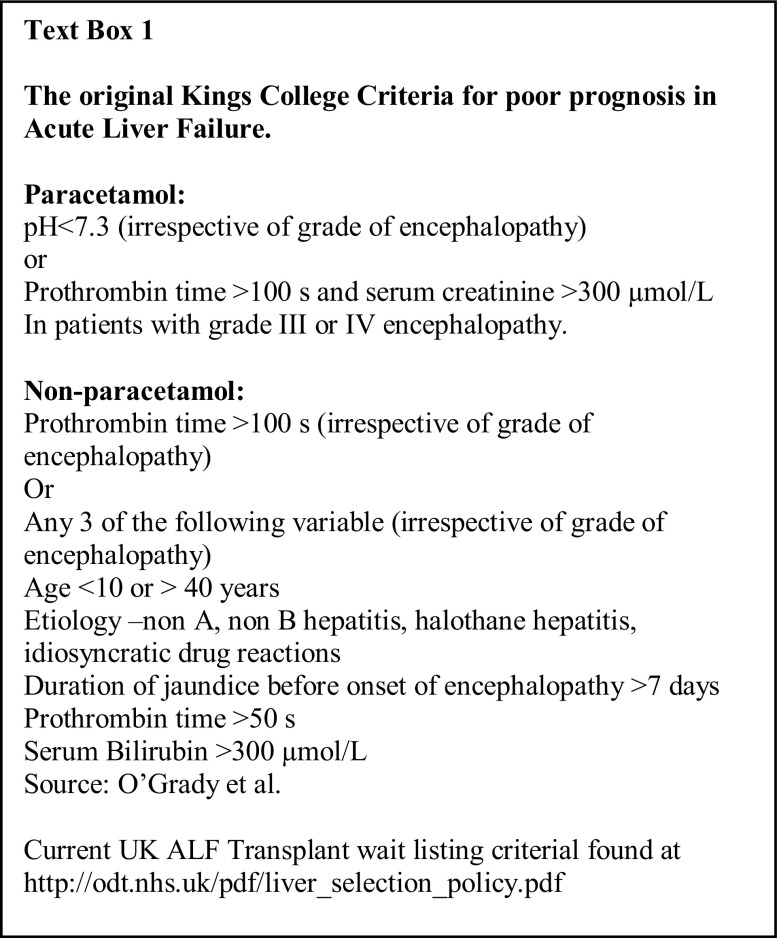


Decision making in relation to the use of transplantation in ALF became even more difficult as improvements in intensive liver care led to higher survival rates with medical management alone. In the King’s College Hospital (KCH) experience of over 3000 patients treated between 1973 and 2008, transplant-free survival had increased to 48%, with a near fourfold increase in overall hospital survival rates—including cases transplanted from 16.7 to 62.2% [4].The increase in ‘transplant-free’ survival was most apparent in paracetamol cases and associated with a substantial fall in the occurrence of cerebral edema and intracranial hypertension, which in the past was often the cause of death in cases with a ‘hyper-acute’ presentation. An important contribution to this improvement is likely through control of circulating levels of ammonia—the principal neurotoxin in this setting—through interventions that include the earlier and more widespread use of continuous hemofiltration [5, 6]. In paracetamol-induced ALF, the narrowing in the gap between survival with medical management alone and survival with transplantation has raised doubts about the transplant benefit in such cases [7]. These changes in the spectrum of complications and improvement in survival have since been documented in other large historical series [8–10].

In this review, we will consider: first, the basis of prognostic assessment systems in ALF and the practical difficulties in their application, with focus upon the KCC as an example of a system in current use. Second, the improved results currently being obtained with liver transplantation in these very sick patients are described and third, how new approaches to treatment may impact upon future care and outcome.

## Improving prediction of outcome

A number of different systems are in current use for the evaluation of prognosis in patients with ALF, with clinical experience of their application and operational performance reported for the KCC and criteria from France and Japan [2, 10–12]. Though details of the models used vary, they share common features (Table 1). All recognize the key importance of the development of encephalopathy as a marker of critically impaired liver function and retain it as a central criterion of poor prognosis. All also utilize laboratory measures of liver function to quantify severity of liver injury, most common measures of coagulation status. Patient Age is also recognized by all as being of prognostic importance—likely reflecting the compromise of physiologic reserve with increasing age—key to surviving any critical illness, and a possible parallel impairment of hepatic regenerative ability. An important difference of the KCC from the other established criteria is the distinction between paracetamol and non-paracetamol etiologies, reflecting the high volume of paracetamol cases seen in the United Kingdom and differences seen in clinical course. Their clinical simplicity has, however, served them well over time, relying not on complex calculation or use of specialized laboratory tests but rather standard bedside assessment and readily available routine laboratory measures.

**Table 1:** Comparison of elements of prognostic scoring systems in current widespread use

Factor	Criteria		
	Kings^b^	Clichy^c^	Japanese^d^
Age^a^	+	+	+
Etiology	+	–	–
Encephalopathy^a^	+	+	+
Bilirubin	−/+	–	+
Coagulopathy^a^	+	+	+

+ Factor included in criteria. − Factor not included in criteria

^a^Factor common to all criteria. References: ^b^[2], ^c^[56], ^d^[57]

Published data illustrate their limitations in current clinical practice, particularly in relation to hyper-acute disease. Here the initial intensity of critical illness may be severe but paradoxically the potential for native liver regeneration is high [4, 9, 11]. In early case series of hyper-acute patients, the dominant clinical issues were those of either a failure to identify all patients with a poor prognosis for consideration of transplantation—limited sensitivity—in concert with that of rapid deterioration of those who fulfilled KCC and high risk of death on the transplant waitlist prior to grafts becoming available, late identification [13–15]. Efforts were, therefore, initially focussed on refining the KCC by improving criteria sensitivity enabling earlier identification of patients with a poor prognosis through integration of supplemental prognostic markers.

Initial studies explored the inclusion of arterial blood lactate measurements determined using point of care testing, as hyperlactatemia reflects both decreased hepatic clearance by a damaged liver and global illness severity and multi-organ failure. Initial reports confirmed their prognostic value, and their inclusion was found to improve specificity, sensitivity and timeliness of the KCC [16]. Other studies have also examined arterial blood lactate in ALF, finding elevated levels to be strongly and independently associated with death or transplantation in both paracetamol and non-paracetamol-induced disease [17–19]. Analysis of the performance of the specific thresholds for arterial lactate introduced into the KCC for paracetamol-induced disease has been less consistent, with one report suggesting that early levels resulted in an increase in sensitivity but at the expense of reduction in specificity [17]. However, two other studies found that lactate measurements alone 12 h after admission to transplant centers had high predictive accuracy, with performance greater than the KCC alone [18, 19]. Though adopted into United Kingdom ALF wait-listing criteria, meta-analysis of validation studies to date has failed to confirm an improvement in diagnostic test performance through their inclusion [20].

**Fig. 1:**
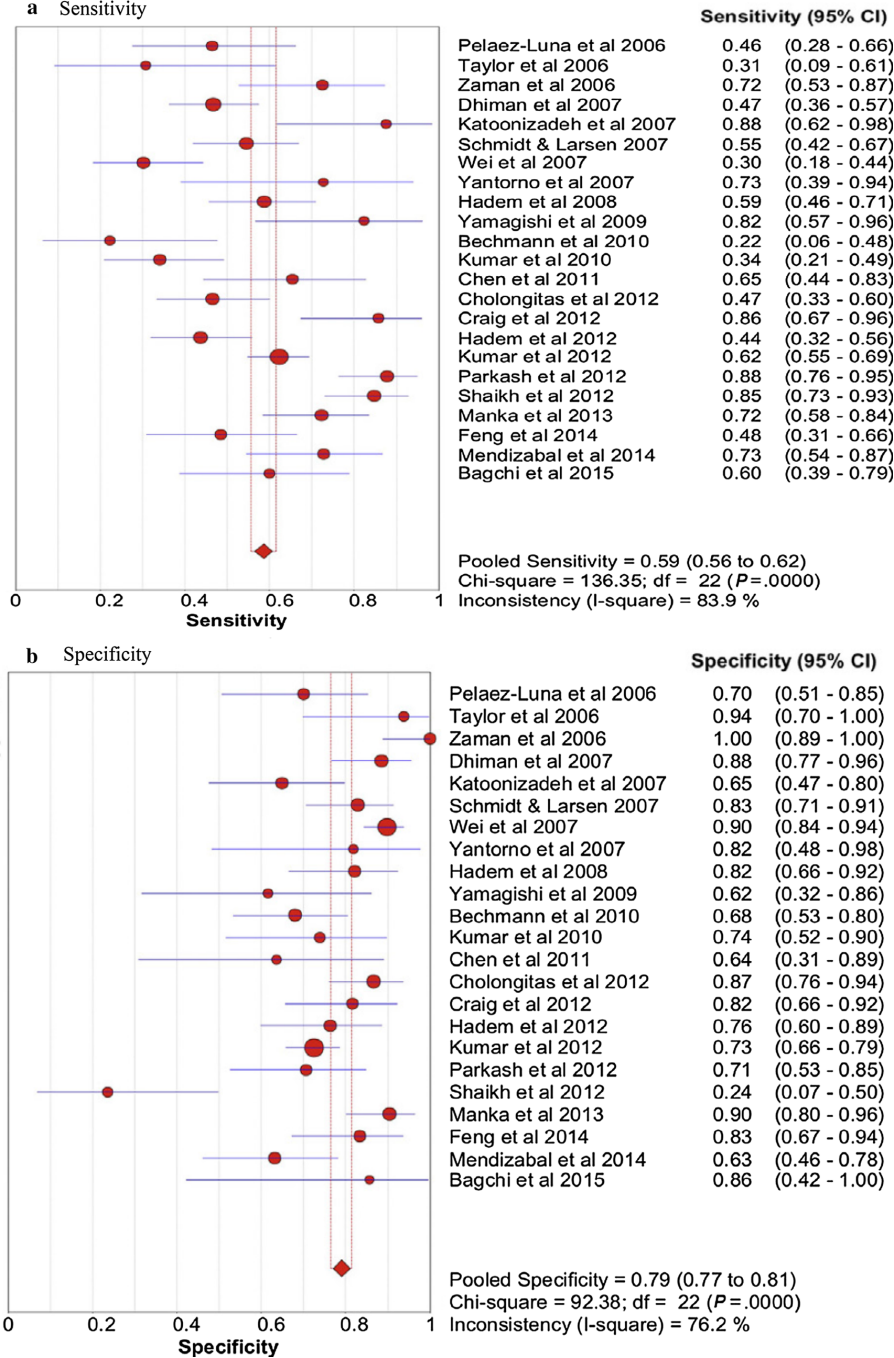
Pooled (**a**) Sensitivity and (**b**) Specificity of Kings College Criteria (Source Ref. [20])

Two further meta-analyses of the performance of the KCC have further illustrated their potential shortcomings (Fig. 1). Review of studies of the KCC in predicting outcome of non-paracetamol-induced ALF comprising 1105 cases, showed overall sensitivity of 68% and specificity 82% [21]. Specificity was highest (93%) in patients with high-grade HE and where the criteria were sequentially determined through the clinical course of illness. Importantly, this analysis also described a fall-off in diagnostic performance in more recent studies, reflecting the increased success of non-transplant approaches to care. A second, larger meta-analysis examining criteria performance in both paracetamol and non-paracetamol etiologies suggested an overall sensitivity of 59% and specificity of 79%, and with better performance values for prediction of non-survival in paracetamol than in non-paracetamol etiologies [22].

Indications are, therefore, of particular aspects where diagnostic performance of selection criteria could be improved beyond existing systems. First, they should consider etiology of illness and reflect the differential in current outcomes of medical care alone. Second, they would perform sequential rather than single time point estimations of prognosis, a process identified on meta-analysis as having additional diagnostic value and reflecting the rapidly changing clinical condition seen particularly in paracetamol and other hyper-acute etiologies and already utilized in other novel prognostic criteria [23]. Recent studies suggest that in these cases waitlist improvement rather than deterioration may now principally confound decision making [9, 11, 12, 15]. Additionally, any new or supplemental criteria should ideally retain the clinical simplicity of existing systems. Two recent attempts to address these issues are discussed below, both of which have utilized the recent availability of web- and app-based computing power to deliver outcome predictions through sophisticated algorithms.

## Dynamic outcome predictive model for paracetamol-induced ALF

**Fig. 2:**
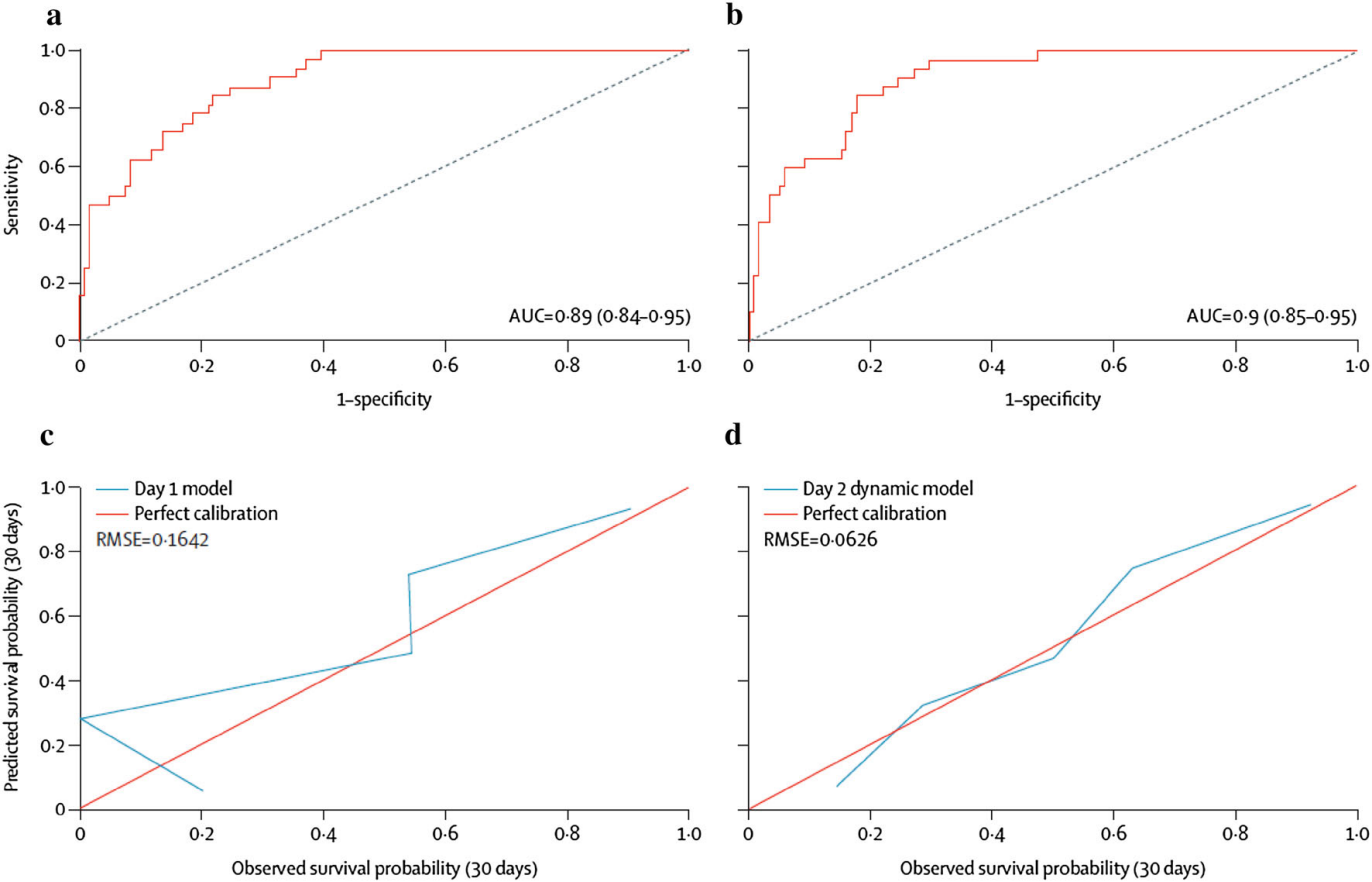
Discrimination and calibration of dynamic prediction model on day 1 and day 2 of admission in 150 patients with paracetamol-induced ALF. **a** Area under receiver operating characteristic curve (AUROC) for day 1 model, **b** AUROC for day 2 model, **c** Calibration curve for day 1 model, **d** Calibration curve for day 2 model (Source Ref. [21])

Prediction models in hyper-acute disease would be of most benefit if they could be applied sequentially identifying and quantifying both improvement and deterioration [22, 23]. The latest model from the King’s Liver Intensive Care Unit is a dynamic outcome prediction model developed and validated for use in patients with paracetamol-induced ALF [24]. It is based on prospective data including analysis of more than 20 daily variables sequentially assessed for 3 days after ICU admission in 912 un-transplanted patients between 2000 and 2012. The variables included in the final models to predict death-included age, hepatic encephalopathy, cardiovascular failure, INR, creatinine and arterial pH on admission and dynamic variables of changing arterial blood lactate and INR. On validation in independent datasets from four transplant centers, the models showed good discrimination between survivors and non-survivors, improving with the inclusion of changes in INR and Lactate over time (Fig. 2). Innovative in this approach was its access though a dedicated website and the generation of continuous survival estimates rather than a binary survival outcome, with the intention that the model should act as a decision-support tool to support clinical judgement rather than a sole arbiter as to proceeding with transplantation.

## Model of acute liver failure study group (ALFSG)

**Fig. 3:**
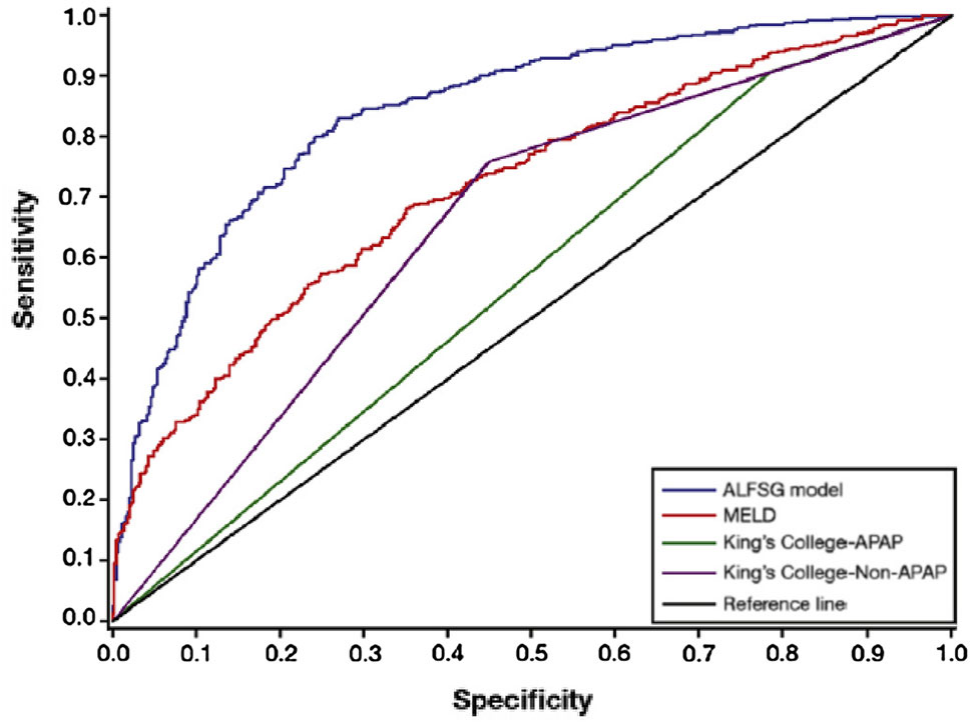
Comparison of the ALFSG model with the Kings College Criteria and MELD score in predicting survival. *ALFSG* acute liver failure study group, *MELD* model for end stage liver disease *APAP* acetaminophen (paracetamol). AUROC: ALFSG: 0.843, MELD: 0.717, KCC APAP 0.560, KCC non-APAP 0.655 (Source Ref. [22])

Elements of this approach were also adopted in the recent analysis by the ALFSG of 1974 subjects enrolled prospectively from 28 academic centers across North America between 1998 and 2013, and managed with and without transplantation [25]. The aim was to devise a mathematical model for all etiologies of ALF to predict transplant-free survival at 21 days, rather than mortality as adopted in other models—making comparison with other studies adopting more standard approaches more complex. Clinical features and laboratory values were collected at study enrolment and recorded serially up to 7 days. Variables of prognostic value adopted in the predictive model included admission coma grade, etiology and vasopressor requirement, and admission bilirubin and INR values. Arterial blood lactate was not explored. In this analyses of both paracetamol and non-paracetamol cases, sequential values of the INR did not add to prediction over that on admission. Using AUROC analysis, test discrimination appeared superior to the KCC and MELD scores (Fig. 3) with the model correctly predicting outcome of illness in 66.3% of subjects. Its performance was best in patients with unfavorable etiologies and high-grade encephalopathy and more limited in those with more favorable etiologies and high-grade encephalopathy.

Though sharing similarities in their use of web-based predictive algorithms, key differences exist between these two predictive systems. One is based on the prediction of mortality and the other survival, with resultant differences in test performance. Further, they are targeted at different patient groups—with the USALF criteria taking a broad approach to all etiologies of ALF, whilst the Dynamic Model is focused upon liver injury from paracetamol where the USALF performs least well. It may be that application of these and other models are complimentary and decision making is based upon the results generated from several prognostic systems [23–25].

## Other systems evaluated

A variety of other scoring systems and supplemental markers have been proposed to identify candidates for liver transplantation, though in general their performance has been evaluated in patient cohorts of limited size (Table 2). Scores used in this way include non-liver specific ones such as SOFA and APACHE II that are widely used to quantify severity of multi-organ failure in other forms of critical illness [17, 26, 27]. Reports suggest similar sensitivity and specificity to the King’s criteria in predicting death and they can be applied sequentially. However, though they may identify those patients at risk of death, the multi-organ failure they quantify may not be corrected by transplantation, if for example, it results for other ALF-associated complications such as severe sepsis or pancreatitis. Consequently, they are not in widespread use to select candidates for transplant.

**Table 2:** Features of illustrative systems for mortality prediction in acute liver failure

Study	Publication year	Criteria	Era	Etiology	*n*	Non-survivors^a^	Sensitivity	Specificity	AUROC	Comment
McPhail et al. [20]	2016	KCC	2001–2015	All	2153	Not Given	0.59 (0.56–0.62)	0.79 (0.77–0.81)	0.76	Meta-analysis
Cholongitas et al. [25]	2012	SOFA	1993–2010	Paracetamol	125	58 (46%)	0.67	0.80	0.79	SOFA Score threshold 12
McPhail et al. [20]	2016	MELD	2001–2015	All	2153	Not given	0.74 (0.71–0.77)	0.67 (0.64–0.69)	0.78	Meta-analysis
Bechmann et al. [29]	2010	MELD/CK18	2006–2009	All	68	18 (27%)	0.85 (0.69–1.0)	0.76 (0.6–0.91)	0.94	Peak M65 fragment
Koch et al. [22]	2016	ALFSG	1998–2013	All	1974	987 (50%)	Not given	Not given	0.84	Prediction of survival not death
Rutherford et al. [30]	2012	ALFSG/CK18	1998–2011	All	500	251 (50%)	0.86	0.65	0.82	Admission M30 fragment
Antoine et al. [32]	2012	Acetylated HMGB1	Not Given	Paracetamol	78	27 (35%)	Not given	Not given	0.87	Admission values

*KCC* Kings College Criteria, *SOFA* sequential organ failure assessment, *MELD* model for end-stage liver disease, *CK18* cytokeratin 18, *ALFGSG* Acute liver failure study group,* HMGB1* high-mobility group box-1

^a^Non-survivors; cases who died or were transplanted. Figures in parentheses are 95% Confidence Intervals where given

The MELD score is more focused on severity of liver injury and has been assessed more widely in ALF and subjected to meta-analysis [22, 28, 29]. In its unmodified form, it shows most promise in non-paracetamol etiologies, with diagnostic performance close to that of the KCC [22]. It has also been combined with circulating blood levels of cytokeratin K18 (CK18) a cell death-associated marker measured using the M30 assay which principally reflects apoptotic cell death [30]. Also, this measurement in place of bilirubin to give a modified MELD Score showed superior sensitivity and specificity to the standard MELD Score and the King’s criteria in predicting outcome of ALF [31].

Other studies have also reported prognostic models combining standard clinical variables with non-standard analytes. By example, the ALFSG index predictive model combined Coma-grade, INR, bilirubin and phosphorus levels with that of blood levels of M30 CK18 [32]. In a 250-strong validation cohort, discrimination between survivors and non-survivors was greater than with the KCC or MELD, though later external validation in a smaller independent cohort showed no advantage over SOFA or APACHE scoring [32, 33].

However, the primary mechanism of liver cell injury may vary by etiology and necrosis rather than apoptosis may predominate. Antoine and colleagues assessed circulating levels of a panel of cell death markers in 78 patients with paracetamol-induced ALF [34]. They found that best prediction of non-survival was with acetylated HMGBI—a biomarker of hepatic necrosis and cellular immunological activation, and to lesser degree with biomarkers of apoptosis including molecular forms of CK18. To date, validation studies have not indicated prognostic advantage above the standard KCC in predicting survival [35]. Cell death biomarkers do appear to be exquisitely sensitive in predicting clinically significant liver injury very early after paracetamol overdose but If they are to be adopted as adjunctive measures to select liver transplant candidates with ALF, key practical issues will need to be overcome [36, 37]. Techniques for their rapid determination will need to be widely available, and it is likely that a panel of biomarkers will need to be assessed to cover etiology-specific differences in the mechanisms of liver cell injury and death.

**Fig. 4:**
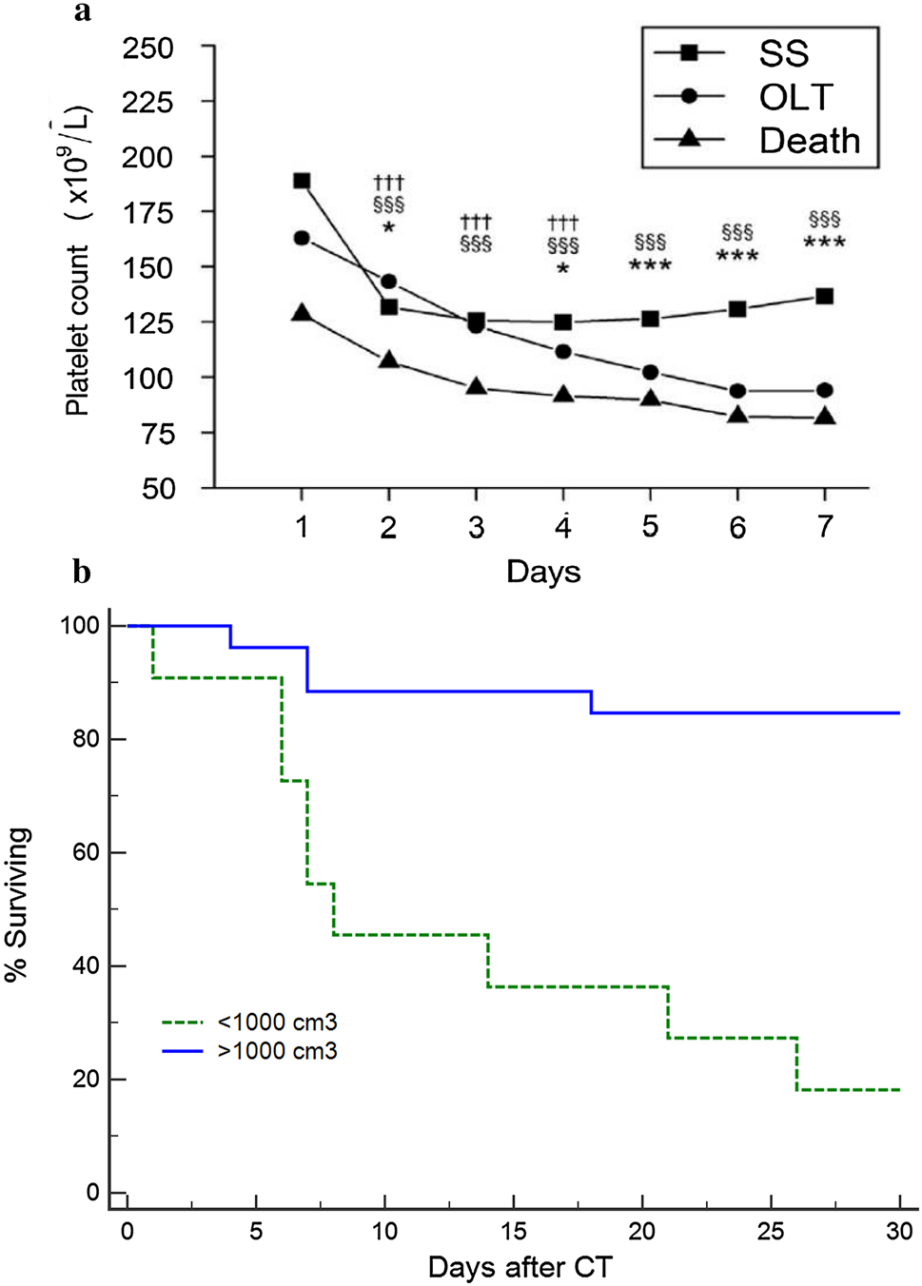
**a** Platelet Counts on days 1–7 after admission in 1598 patients with acute liver failure according to outcome at 21 days. *SS* spontaneous survivor, *OLT* orthotropic liver transplant. Three symbols, *p* < 0.001; two symbols, *p* < 0.01; one symbol, *p* < 0.05. *SS vs LT, ^§^SS vs death, and ^†^LT vs death. SS, spontaneous survival. **b** Survival According to CT-derived liver volume on admission in 37 non-transplanted patients with non-acetaminophen etiologies. < 1000 cm^3^ *n* = 11, ≥ 1000 cm^3^ *n* = 26, *p* < 0.001 log-rank (Source Ref. [36, 38])

Other simpler clinical measures have also demonstrated potential for use as prognostic markers: platelet count has been shown to be closely linked to outcome. In a recent study from the USALFSG, the evolution of thrombocytopenia was closely associated with development of multi-organ failure and a poor outcome in ALF and linked to the development of a systemic inflammatory response [38] (Fig. 4a). Liver volume may be easily determined using analysis of CT images and reflect the balance between the parallel processes of liver collapse with cell death and increase with regeneration. Recent studies suggest that in some non-paracetamol etiologies, loss of liver volume in adults to less than 1000 cm^3^ may indicate irreversible damage and serve as an early indicator of poor prognosis, often in advance of the development of encephalopathy (Fig. 4b) [39–41].

Other studies have also sought to identify at a very early stage those patients with acute liver injury (ALI) in the absence of encephalopathy which will later progress to a poor outcome of death or transplantation. In a study of US patients hospitalized with ALI, progression to a poor outcome was more common in non-paracetamol etiologies and with higher levels of INR and bilirubin, and a prolonged duration of illness [42].

Finally, it is important to emphasize that prognostic models should be only part of the overall functional evaluation of the very sick patient with ALF and an experienced multi-disciplinary team in an intensive care setting is required for correct interpretation. Rather than providing an absolute arbiter, these models should support decision making and the multifactorial team assessment.

## Current results of liver transplantation

Life-saving though it was soon seen to be after its introduction, overall results not surprisingly for these very sick patients were significantly lower than for elective transplants carried out for those with chronic diseases. Several series have now demonstrated a progressive incremental improvement in survival over time, with patient survival now approaching that of elective surgery. Recent reports for the US and UK are of 1-year patient survival rates of 85–90% [43]. These results reflect advances in peri- and intra-operative care and the interplay of several factors: the earlier and more accurate prediction of need for transplant and identification those patients who are too sick for surgery, and a better understanding of the interplay between graft and recipient factors in determining patient survival. Analysis of the European experience of 4903 transplant recipients between 1998 and 2009 found death after surgery to relate to both with independent association shown with male recipients (adjusted OR 1.25), recipient > 50 years of age (1.26), incompatible ABO matching (1.93), donors > 60 years (1.21), and reduced size graft (1.54). Recipients > 50 years, combined with donors > 60 years had a 57% mortality/graft loss within the first year after transplant [44]. Matching of graft to recipient is key to ensuring optimal outcome [13]. Case series also now confirms the practicality and excellent outcomes obtained for selected patients transplanted using living donors, and from auxiliary liver grafting in etiologies where native liver regenerative potential exists—though globally, these techniques are applied to only a small proportion of recipients [45, 46].

Patient survival after transplantation for ALF follows a characteristic pattern of increased mortality early after transplant, but in patients who survive this phase, subsequent survival parallels closely with that seen in elective transplantation [44]. In an early series, the high prevalence of psychiatric comorbidity and addiction issues seen in recipients of transplantation for paracetamol-induced ALF was reflected in an increased risk of death due to suicide, trauma or non-adherence to immunosuppression with over half the deaths occurring within 12 months of transplantation [44]. Later series have not demonstrated such an increase in mortality, but it is clear that paracetamol recipients show increased levels of psychotic comorbidity which may be reflected in poor compliance with medication and follow-up [47]. In these patients, assessment of severity of psychiatric illness is a key element of the selection of potential recipients and in those transplanted follow-up must include close ongoing psychiatric monitoring and support.

## New novel non-transplant options

A number of novel non-transplant interventions have potential to serve as alternatives to transplantation. A central premise to their use is that the injured liver retains regenerative ability and that this may be augmented by these interventions. A better practical understanding of the processes influencing both liver injury and regeneration is likely to be of clinical importance here, exemplified by the major clinical and laboratory differences between paracetamol hepatotoxity from single time point or staggered overdoses—and the markedly worse outcomes seen in the latter group [48]. The potentially important influence of pre-existing sub-clinical non-cirrhotic liver disease upon liver regeneration is currently poorly characterized, for example, from chronic alcohol use or non-alcoholic steatohepatitis.

In cases where the liver injury has progressed ‘beyond the point of no return’, novel medical therapies are unlikely to be of benefit. It would thus be of no surprise that most efficacy would be seen either in treatment at an early stage of disease and/or in etiologies with greatest regenerative capacity. By example, the FULMAR trial of the MARS extra-corporeal device in patients with ALF failed to show a survival improvement in the overall study cohort but there were indications of potential benefit in patients with paracetamol-related disease [49].

**Fig. 5:**
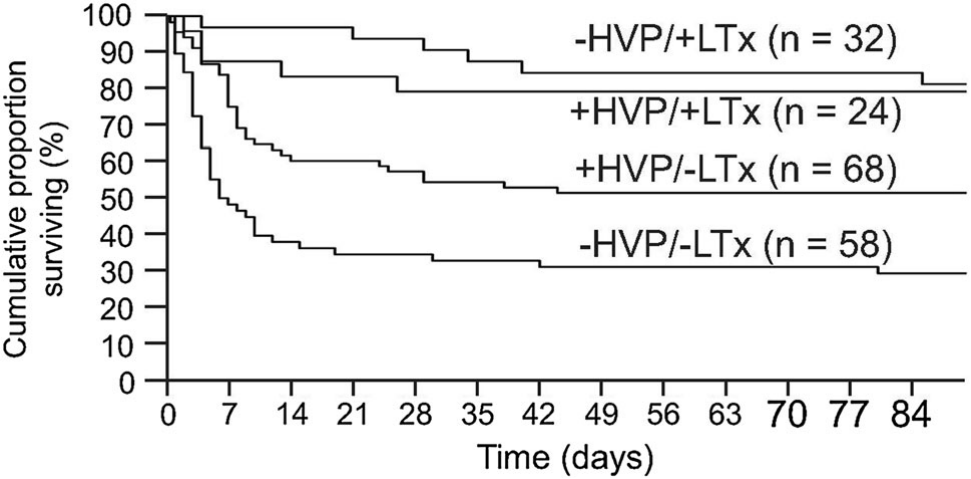
Survival in 182 patients with ALF according to treatment with liver transplantation and high-volume plasma exchange. Two groups receiving SMT (standard medical treated group) with and without emergency transplantation (HVP + LTx vs +HVPLTx) and the two group receiving SMT with and without emergency transplantation (HVPLTx vs. +HVPLTx) (log rank: *p* = 0.0058) and Cox proportional hazard: LTx: *p* < 0.0001; HVP: *p* = 0.0076) (Source Ref. [48])

Such caveats may also apply to the use of therapeutic plasma exchange (TPE). Through mechanisms thought to include removal of deleterious inhibitors of hepatic regeneration and a complex pattern of immunomodulation, use of this therapy has recently been demonstrated to deliver a survival benefit in non-transplanted patients with ALF. In a multi-center randomized controlled trial on mixed etiologies of disease, three 5-l TPE sessions significantly improved survival above that seen with standard medical care, although the survival benefit was inferior to that seen with transplantation (Fig. 5) [50]. There is ongoing debate about the place of this therapy in patients with ALF, given that its use normalizes measures of coagulation disturbance and thus precludes their use in prognostic evaluation for later transplantation. Our practice is in accordance with recent EASL guidance, with its early use in patients with ALF with an expected poor prognosis without transplantation but who have clear medical or psychiatric contra-indications to surgery [51]. In addition, the use of TPE is associated with significant improvements in cardiovascular status and we also treat patients who are waitlisted for liver transplantation but with worsening cardiovascular failure and vasopressor requirement while awaiting a graft [50].

Other forms of cellular and immune-modulatory therapy show promise as future treatments for ALF. Whilst hepatocyte transplantation retains considerable theoretical attractions, it presents considerable logistic challenges and a requirement for immunosuppression, and its benefit is yet to be established in adult patients with ALF [52, 53]. Other interventions in patients with acute-on-chronic liver failure (ACLF) show promise but are yet to be tested in ALF. Understanding of the pathophysiologic basis of ACLF is rapidly increasing and the insights gained, particularly in relation to modulation of regeneration and immunological function, may well be transportable to ALF. In a randomized controlled single-blind trial in 110 patients with Hepatitis B-related ACLF of whom less than half were thought to be cirrhotic, treatment with allogenic mesenchymal stem cells improved survival—[54]. Similarly, in a single-center double-blind trial in 55 patients with decompensated cirrhosis, treatment with granulocyte colony stimulating factor and erythropoietin was associated with improved survival in association with indications of improved hepatic regeneration through mobilization of progenitor cells [55]. These results are yet to be replicated or tested in patients with ALF but demonstrate mechanistic approaches likely to be assessed in the near future. As ever, the rarity and severity of ALF represent major limiting factors for performing randomized controlled trials of these or other agents and true advances in therapeutic intervention are likely to result only from international collaborative studies.
